# Chemical Structure, Sources and Role of Bioactive Flavonoids in Cancer Prevention: A Review

**DOI:** 10.3390/plants11091117

**Published:** 2022-04-20

**Authors:** Georgiana Drețcanu, Ioana Știrbu, Nicolae Leoplold, Daniel Cruceriu, Corina Danciu, Andreea Stănilă, Anca Fărcaș, Ileana Monica Borda, Cristian Iuhas, Zorița Diaconeasa

**Affiliations:** 1 Faculty of Food Science and Technology, University of Agricultural Sciences and Veterinary Medicine, 3-5 Calea Mănăştur, 400372 Cluj-Napoca, Romania; georgiana.dretcanu@stud.ubbcluj.ro (G.D.); andreea.stanila@usamvcluj.ro (A.S.); anca.farcas@usamvcluj.ro (A.F.); zorita.sconta@usamvcluj.ro (Z.D.); 2 Faculty of Physics, Babes-Bolyai University, Kogalniceanu 1, 400084 Cluj-Napoca, Romania; ioanaalexandrastirbu@gmail.com (I.Ș.); nicolae.leopold@phys.ubbcluj.ro (N.L.); 3 Department of Molecular Biology and Biotechnology, Babes-Bolyai University, 5-7 Clinicilor Street, 400006 Cluj-Napoca, Romania; daniel.cruceriu@ubbcluj.ro; 4 Department of Genetics, Genomics and Experimental Pathology, The Oncology Institute “Prof. Dr. Ion Chiricuta”, 34-36 Republicii Street, 400015 Cluj-Napoca, Romania; 5 Department of Pharmacognosy, Victor Babes University of Medicine and Pharmacy, 2 Eftimie Murgu Sq., 300041 Timisoara, Romania; corina.danciu@umft.ro; 6 Sixth Department of Medical Specialties, Medical Rehabilitation, Iuliu Hațieganu University of Medicine and Pharmacy, 400012 Cluj-Napoca, Romania; monica.borda@umfcluj.ro; 7 Faculty of Medicine, Iuliu Hatieganu University of Medicine and Pharmacy, 400372 Cluj-Napoca, Romania

**Keywords:** flavonoids, polyphenols, antioxidants, cancer, genetic manipulation, plant-based diet

## Abstract

There has been a major shift in the collective mindset around the world in recent decades, both in terms of food and in terms of the treatment of chronic diseases. Increasing numbers of people are choosing to prevent rather than treat, which is why many consumers are choosing plant-based diets, mainly due to their bioactive compounds. A significant case of bioactive compound is flavonoids—a wide subclass of an even wider class of phytochemicals: polyphenols. Flavonoids are a broad topic of study for researchers due to their potential in the prevention and treatment of a broad range of cancers. The aim of this review is to inform/update the reader on the diversity, accessibility and importance of flavonoids as biomolecules that are essential for optimal health, focusing on the potential of these compounds in the prevention of various types of cancer. Along with conventional sources, this review presents some of the possible methods for obtaining significant amounts of flavonoids based on a slightly different approach, genetic manipulation.

## 1. Introduction

Plants have been a subject of interest for human beings since the beginning of time. First, the symbolistic nature of plants was described in mythology. Some fruits such as lemons were motifs used in the decoration of cultural monuments, while grapes were considered gifts form the gods because of their nutritional value [1]. After that, in ancient times, people extracted phytochemicals from different medicinal plants because they believed that they had healing properties. As time went on, humanity gradually shifted to a meat-based diet thanks to cultural innovation, but in today’s society, the consumption of plant-based foods has become an increasingly approachable lifestyle [2].

Even though in some cultures, such as Christian culture, plants are considered a gift from God for the purpose of nourishing and treating human on Earth [3], fundamentally, plants do not produce these compounds for humans but for their own purposes. Phenolics are in fact secondary metabolites—bioactive substances with both an antioxidant role and an attractive role for pollinators, while also representing a defense mechanism against ultraviolet radiation and several biological pathogens [4]. 

Polyphenols are a group of phytochemical substances, characterized by the presence of more than one phenol group per molecule [5]. They are classified into different classes such as monophenols, flavonoids, phenolic acids and other non-flavonoid polyphenolics. Even though each subgroup of polyphenols has some well-studied and frequently used representatives, what has gained specific attention from the scientific world are the flavonoids, which are highly diversified pigments that occur ubiquitously in nature. There are several thousands of flavonoids, representing one of the largest groups of naturally occurring products. Flavonoids are widely distributed in plants and are easily recognized as flower pigments, but they occur as well in all parts of the plant.

As the structure of these compounds became increasingly complex and versatile, flavonoids acquired new “responsibilities”, becoming able to manipulate not only the plant cell but also the animal cell. In other words, once the human species began consuming plants, they used these biocompounds for their own purposes to maintain their antioxidant balance and to protect their genetic material, proteins and lipids from possible mutagenic factors, whether internal or external. Of course, during the evolution of science, not only were the beneficial effects of these compounds demonstrated, but also some toxic effects were encountered at the cellular level. If we refer to a normal, healthy cell, cases of toxicity can be observed only in the case of overdoses or the consumption of huge quantities of plant-based foods. On the other hand, if we refer to a modified cell, such as a cancer cell, the phenomenon of inducing toxicity is desirable, because prevention and treatment schemes aim to reduce tumor development. Numerous studies have shown that flavonoids exert pro-oxidative properties on cancer cells, which has intrigued the scientific community worldwide and brought the subject of flavonoids into another perspective: the possible antitumor compound used in prevention/treatment regimens. As phytochemical compounds, their sources of procurement are diverse, so the premises that state that flavonoids can be a good agent for cancer prevention are numerous [6].

In the next chapters, flavonoids will be presented from different perspectives, starting with a short chemical characterization, continuing with some of the richest sources of flavonoids and ending with their implication in cancer prevention.

## 2. Methods

This paper is an overview of the chemical structure, sources and role of bioactive flavonoids in cancer prevention.

The literature search took place in the PubMed, Web of Science, Scopus and the academic search engine Google Scholar databases. The following keywords were used: flavonoids* AND cancer prevention, flavonoids* AND classification, flavonoids* AND rich dietary sources and flavonoids* AND genetic and metabolic. The results were screened based on their titles, abstracts and full-text availability. All non-English publications were excluded from the present review. Filter limits (such as text availability, article type and publication date) were not applied.

## 3. Chemical Structure and Classification of Flavonoids

Flavonoids are polyhydroxy-phenolic compounds of the phenylpropanoid biosynthetic pathway in plants [7,8]. They present 15 carbon atoms (C_6_-C_3_-C_6_) that build the structure of two benzene rings joined by a heterocyclic oxygen-centered ring, constituting one of the most characteristic classes of compounds in higher plants. In fact, these compounds are the best-known group of phenolics of mixed biosynthetic origin, where the A ring is synthetized in the polyacetate pathway, the B ring is synthetized in the shikimate pathway and the C ring comes from both of these pathways, as a condensation product of secondary metabolites [9,10]. Many studies have demonstrated common features of flavonoids that are highly important to their different activities, such as their planar structure, the number and position of their substituent groups as well as the presence of the C_2_-C_3_ double bond [11].

**Figure 1:**
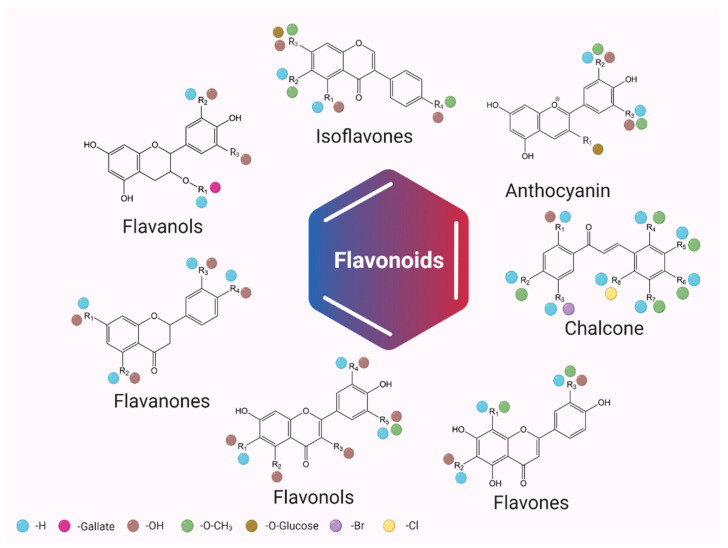
Flavonoids classification and their possible chemical structure (created with BioRender.com, accessed on 6 April 2022).

Flavonoids are classified into various subclasses, based on the substitution patterns of ring C, the oxidation state of the heterocyclic ring and the position of ring B. Therefore, flavonoids are divided into seven major subclasses: flavan-3-ols, flavones, flavonols, flavanones, anthocyanins, chalcones and isoflavonoids (Figure 1). Flavanones, flavones, flavonols, flavan-3-ols and anthocyanins present ring B in position 2 of the heterocyclic ring, and isoflavonoids present ring B in position 3. Flavanones and flavan-3-ols have the central heterocyclic ring saturated and, in this case, one or more chiral centers are present. On the other hand, anthocyanins, isoflavones, flavones and flavonols have the central heterocyclic ring unsaturated, with the molecule being achiral [11]. In the case of chalcones, they represent the only subclass that has a unique structure: it is a little different than usual, but it exerts the same properties. They are precursors in flavonoids and isoflavonoids biosynthesis, having two aromatic rings joined by a three-carbon α,β-unsaturated carbonyl chain. They are found both in the form of cis and trans-isomers, in contrast to the rest of the flavonoid subclasses [12].

As shown in Figure 1, each subclass of flavonoids has different and unique sets of substituents bonded with different carbon atoms, the main ones being -H, -OH, -*O*/*C*-Glycoside, -Mannoside, -Galactoside, -Methyl, -Gallate and -Acyl groups. The majority of them can be found in the primary structure of flavonoids in planta (e.g., in fruits, the most stable form of flavonoids is flavonoids-*O*-glycoside) [11]. The rest can obtain these new substitutes during metabolic processes in the gastrointestinal tract (e.g., in hepatocytes, the main reactions that can occur as part of the phase II metabolism are methylation, glucuronidation and sulphonation) [13].

**Figure 2:**
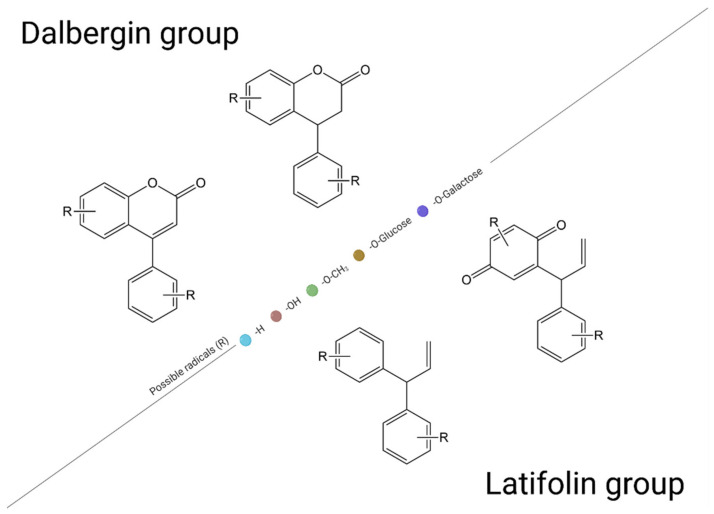
Neoflavonoids classification (created with BioRender.com, accessed on 6 April 2022).

Although established, these compounds are not the only compounds of this kind produced in the plant kingdom. Recent studies have found so-called neoflavonoids—compounds similar to classical flavonoids but that have certain peculiarities. Neoflavonoids are not produced very often by edible plants but by a variety of plants belonging to families such as Fabaceae, Leguminosae, Rubiaceae, Passifloraceae or Polypodiaceae. Another particularity is that neoflavonoids are also classified into two groups—the dalbergin group (4-phenylcoumarins) and latifolin group (diphenyl allyl compounds)—but the classification depends on the pattern of substitution and on sources. They can present different configurations, as shown in Figure 2, depending on the nature of radicals attached (e.g., -OH, -Glycoside, -Galactoside, -Rhamnoside), and, because of this versatility, they can also be utilized in prevention and treatment schemes, just like flavonoids. It is already known that neoflavonoids exert interesting properties, such as cardiovascular, antidiabetic, antioxidant, antiplasmodial, anti-inflammatory, anti-allergic, anti-melanogenic, antimicrobial, anti-osteoporosis and antileishmanial activity, but also cytotoxic activity against several cancer cell lines [12]. However, because of the fact that neoflavonoids are not considered dietary biocompounds, they are not the subject of this review.

## 4. Rich Sources of Flavonoids

### 4.1. Berries and Fruits

Berries seem to capture increasing attention from the general consumer, with both the demand and the supply on the market being in a continuous growth. They possess a wide range of health benefits, the most known being their antioxidant properties, which are strongly related to their high content in flavonoids. Certainly, the phytochemical content varies among cultivars, location, harvesting period and environmental factors, but these variations precisely give the body the biochemical diversity it needs in order to function optimally [14]. 

Statistically, among all berry-type fruits, blueberries and lingonberries have been shown to contain the highest amounts of flavonoids (1100 mg/100 g dry weight-DW), followed by raspberries and strawberries (500 mg/100 g DW), but many other berries have been reported to be rich in flavonoids as well [15,16]. Besides their high concentration in berries, their chemical properties help them to keep both their stability and biological functions intact, also in products resulting from biotechnological processes. A good example is grapes and their derivatives, such as juices and wines, which also possess a large variety of flavonoids [17]. Studies have shown that wines can reach 2.2 mg/L of total flavonoids [18], and grape juice (verjuice) can reach 2.6 mg rutin equivalent (RE)/mL total flavonoids [19].

From another perspective, a number of researchers have focused on the narrow frame, wanting to find out exactly which compounds give berries, but also fruits, these antioxidant capabilities, thus demonstrating that, for the most part, the most impactful flavonoids in berries are anthocyanins and flavonols (Table 1) [20,21,22,23].

Anthocyanins are naturally occurring pigments responsible for the red, blue and purple colors of fruits. Therefore, the most intense-colored berries are those that possess the highest content of anthocyanins. Various studies have reported the usefulness of anthocyanins. Not only do they serve as nutraceuticals, but they are also considered functional food ingredients, as they are widely used as natural colorants in the food industry [36]. The major compound from the anthocyanin subclass found in berries is cyanidin, but it is found mostly in a glycosylated form, due to its higher stability in the acidic nature of the berries [37].

Anthocyanidins and anthocyanins’ aglycones, such as delphinidin and petunidin, are found in high amounts in blueberries (about 27 and 28 mg/100 g FW), while pelargonidin is dominant in strawberries (about 347 mg/100 g FW) [25].

Other abundant flavonoids found in berries are the compounds from the flavonols subclass. Flavonols are yellow pigments that contain a double bond between C2 and C3 and a -OH group in position 1. As anthocyanins, flavonols are also found in berries in a glycosylated form, usually linked to a glucose or rhamnose molecule [18]. Among all others, quercetin and kaempferol are the most encountered flavonols in almost all berry-type fruits [38,39,40,41,42,43].

Besides berries, citrus fruits are also a great dietary source of bioactive compounds. Flavonols, flavones and flavanones are present in all citrus fruits, known as strong free radical scavengers. Flavanone-*O*-glycosides, flavone-*O/C*-glycosides and their derivatives were found to be the most abundant flavonoids in genus *Citrus*. Naringenin and hesperidin, the main compounds belonging to flavanones subclass, have been reported in citrus fruits, and they are responsible for the bitterness of citrus juices and peel. A study conducted on various fruits and vegetables, including citrus fruits, showed that naringenin and hesperidin were identified in high contents in citrus [29]. Hesperidin was present in higher concentrations in lemon (17 mg/100 g FW), lime (43 mg/100 g FW) and orange (31 mg/100 g FW), while in grapefruits, naringenin had a higher concentration (53 mg/100 g FW) [29].

### 4.2. Vegetables

Flavonoids represent a significant proportion of the total polyphenol content identified in vegetables, although they are not considered a source of phenolic compounds as rich as fruits. Some of the richest sources of flavonoids include radish (45 ± 1.24 mg catechin equivalents (CE)/100 g FW) and spinach (29 ± 1.24 mg CE/100 g FW), followed by pepper (25 ± 1.63 mg CE/100 g FW), potato (18 ± 0.47 mg CE/100 g FW) and onion (17 ± 2.16 mg CE/100 g FW) [44]. 

While flavonols are found mostly in bell peppers, chili peppers and lettuce, flavanones are mostly found in tomatoes. Yellow bell pepper is found to contain about 10.2 mg/100 g DW quercetin, 9.5 mg/100 g DW luteolin and a total of 19.8 ± 0.4 mg/100 g DW flavonoids. Green pepper, on the opposite pole, contains only 7.1 ± 0.1 mg/100 g DW quercetin, 6.2 ± 0.5 mg/100 g DW luteolin and a total of 13.7 ± 0.6 mg/100 g DW flavonoids, being the poorest in flavonoids among all the sweet peppers [45,46]. Some other flavonols, such as kaempferol (4.13 ± 0.24 mg/100 g FW) and isorhamnetin (5.3 ± 0.04 mg/100 g FW), were found in high concentrations in vegetables such as onions [30,47]. 

Flavones such as apigenin and luteolin were identified in vegetables such as kale, radish, celery and cabbage [30], and some considerable amounts of anthocyanins and their aglycones were identified in red onion, purple kale, red radish, red cabbage, purple sweet potato and red cabbage, with cyanidin-glycosides being the main anthocyanins identified (Table 2) [48].

### 4.3. Spices

Spices and herbs have been widely used in traditional medicine due to their beneficial properties for human health. Several reports have shown that spices and herbs are valuable sources of natural phenolic antioxidants. More than that, spices have been shown to possess much higher antioxidant properties than fruits and vegetables, which were correlated with the total phenolic content. Flavonoids are one of the major phenolics in spices, and they generally occur as glycosylated derivatives [52].

In the U.S. Department of Agriculture (USDA) database (2014), it is suggested that parsley has the highest total flavonoid content (4845.5 mg/100 g), followed by Mexican oregano (1550.79 mg/100 g), celery seeds (841.05 mg/100 g) and Tasmanian pepper (752.68 mg/100 g). Capers also have a high content of flavonoids (493.03 mg/100 g), with saffron (205.48 mg/100 g), dill (112.68 mg/100 g), thyme (47.75 mg/100 g) and rosemary (27.41 mg/100 g) being also in the top list. The spice with the lowest flavonoid content is garlic, with only 3.61 mg/100 g [57].

The main subclass of flavonoids found in spices is flavones (Table 3), with apigenin and luteolin being present usually in aromatic herbs, such as parsley, rosemary, oregano, basil and thyme [52]. Peppermint is a good source of flavanones such as eriodictyol (12.27–54.53 mg/100 g FW) and hesperetin (21.94 mg/100 g FW) [26]. Flavonols such as quercetin and kaempferol were identified in coriander, caraway, oregano, basil, dill and parsley. Other flavonols such as myricetin, rutin and isorhamnetin were identified in different spices. Some of these flavonoids represent the active substance in spices. Therefore, apigenin is the active substance in parsley, luteolin in oregano and celery and kaempferol in capers [58].

### 4.4. Genetically Modified Organisms

Although most studies acknowledge dietary foods as the main source of phenolic compounds, it is important to recognize that, in order to benefit from the properties of these compounds, relatively high concentrations of the active compound must be consumed, which cannot be obtained only through the consumption of wild-type plants. Thus, some researchers have approached various techniques of bacterial DNA recombination or genetic engineering and editing of plants in order to manipulate the amounts of flavonoids produced.

#### 4.4.1. Plant Genetic Engineering and Editing

Plant genetic engineering is a technique that integrates a desired DNA fragment (recombinant DNA) into another organism’s genome, using basic knowledge of molecular biology. Once this technique is performed, it will result in an improved plant organism, which will perform new functions, produce smaller or larger amounts of compounds or gain some resistance or sensitivity to biotic or abiotic factors, depending on the genes of interest introduced into the body [59].

An interesting approach to producing higher concentrations of flavonoids is the genetic transformation of hop plants via *Agrobacterium tumefaciens*, which contains an *Arabidopsis thaliana* regulatory factor construct, such as the production of anthocyanin product 1/*A. thaliana*’s MYB transcription factor 75 (PAP1/AtMYB75). The transgenic hop plants were reported to have higher concentrations of anthocyanins, rutin, isoquercetin, kaempferol-glucoside, kaempferol-glucoside-malonate, desmethylxanthohumol, xanthohumol, a-acids and b-acids than wild-type plants. Furthermore, the same technique was used successfully for the production of purple tomatoes, cauliflower and rice and red apples with enhanced anthocyanin content [60].

Another study focused on plasmid-mediated transformation via *Agrobacterium* and was performed by Reddy et al. [61]. They used a rice callus culture that they transformed with *Agrobacterium* by inserting a plasmid construct into callus cells. The plasmid contained the complementary DNA (cDNA) of the enzyme anthocyanidin synthase (ANS) under a constitutive promotor of mannopine synthase (MAS) (*Pro_MAS_: ANS)*. After the transformation and regeneration, the transgenic plant exhibited an increased antioxidant activity due to the higher levels of anthocyanins and flavonols. These results were obtained because the rice transgenic plant expressed higher levels of ANS, which not only increased the concentrations of anthocyanins and quercetin specifically, but also decreased the proanthocyanidin level in a tissue-specific manner [61]. 

Similar results were obtained by Schijlen et al. [62], who used a double promotor with constitutive cauliflower mosaic virus double 35S promoter (Pd35S), a gene encoding chalcone isomerase (CHI), a gene encoding flavone synthase (FNS) and *Agrobacterium tumefaciens nos* terminator (Tnos) for tomato plant transformation. After the transformation, it was observed that tomato peel accumulates higher levels of flavonols such as luteolin aglycone (up to 340 mg/kg FW) and luteolin 7-glucoside (up to 150 mg/kg FW) than the wild-type plant [62].

From other perspective, some researchers managed to enhance flavonoid concentration by mutating (e.g., insertion, deletion, substitution) specific loci in the whole genome. This is called genome editing and is performed especially through specialized constructs of microbiological origin, called Clustered Regularly Interspaced Short Palindromic Repeats (CRISPR) and CRISPR-associated proteins (Cas) [59]. Zhang et al. [63] tested this novel technology on soya plants (*Glycine max*) for the purpose of increasing isoflavones content in soya beans. A CRISPR-Cas9 construct was designed to induce triple mutations in order to knock out the genes for flavone synthase (*GmFNSII-1*) and flavanone-3-hydroxylase (*GmF3H1* and *GmF3H2*). These two enzymes compete with isoflavone synthase (IFS) for naringenin (the substrate). After the editing, mutant soya beans presented a higher level of isoflavones, especially genistein (more than twice the concentration compared to the wild-type), and, because of the higher production of isoflavones, soya leaves presented resistance against soya bean mosaic virus (SMV) [63,64].

#### 4.4.2. Bacterial DNA Recombination

Flavonoids can also be obtained in an alternative way—a path little approached by researchers in the field but that provides excellent results in terms of the quantities obtained. This is also a topic focused on metabolic engineering, but it is not about genetic editing—it is about the recombinant DNA technology which makes the bulk production of different types of flavonoids into a bacterial cell possible, which is an advantage because high concentrations of the compound can be supplied quickly and can be used later for curative purposes. An interesting experiment was performed by Watts and his team [65], who obtained 20.8 mg/L of naringenin in 48 h in recombinant *Escherichia coli* cultures. They induced cultures of *E. coli* with genes from *Arabidopsis thaliana* that would translate the enzymatic package needed for naringenin synthesis [65]. Therefore, the structure of flavonoids is really an advantage, not only in terms of the healing purposes they exhibit but also because of the quick and easy methods of obtaining them in natural and biotechnological ways. 

Another similar study made by Lyu et al. [66] revealed the vast possibilities for the production of naringenin in *Saccharomyces cerevisiae*—a yeast strain. *S. cerevisiae* was transformed with constructs that summed up the genes responsible for the production of enzymes from the naringenin biosynthetic pathway. They managed to produce about 90 mg/L naringenin from tyrosine (amongst the highest possibilities for de novo microbial production), using shake flask fermentation, which again demonstrates the potential of genetic manipulation in phenolics mass production [66].

## 5. Flavonoids and Human Health

Phytochemicals have been used for centuries in the production of medicines or foodstuffs whose main purpose was to maintain the health and integrity of the individual [67]. Recently, flavonoids have been the subject of considerable scientific and therapeutic interest, because these natural functional compounds can serve as a starting point for the development of optimal drugs [68].

The advantages of these compounds are numerous, but the most important assets worth considering are their wide distribution, their great structural variety and their low production costs. Furthermore, flavonoids are small organic compounds thats are easily metabolized and absorbed by the human body, and because of that, they could be one of the safest non-immunogenic drugs used in the pharmaceutical industry. In fact, there are many expectations that a wide range of diseases can be successfully treated with newly developed nanoformulations of flavonoids or their derivatives in the near future, since the therapeutic applications of flavonoids normally do not trigger immune reactions [69].

Their use for pharmaceutical purposes is supported by their chemical structure, which makes them responsible for a variety of pharmacological activities. This polyphenolic structure is achieved due to the enzymatic packages held by each plant species, given that flavonoids are, as mentioned above, secondary metabolites in multiple metabolic pathways. It is worth mentioning that, for a mass use of these compounds, enormous concentrations are needed, which can be obtained both naturally and biotechnologically [69].

Flavonoids can be exploited directly from the source through the foods that make up the daily diet or through the extraction of flavonoids and the use of the concentrate in the production of administered nanoformulations. Although the diet method seems to be the easiest one, the problem is that the low bioavailability of flavonoids is an impediment in the absorption of a high concentration of the active compound. Low bioavailability refers to the fact that, once ingested, the compound reaches the systemic circulation with difficulty, resulting in a low rate of cellular absorption due to the high rate of metabolism and poor solubility [70]. That is the reason why increasing numbers of researchers have focused their attention on enhancing the flavonoid concentration and/or on patenting the encapsulation methods by which relatively high amounts of bioactive compound can be transported and protected throughout the body via polymeric coats or diverse matrices and can be transported to target cells [71].

In terms of health properties, many studies have demonstrated their various biological activities including anti-inflammatory, anticancer, antibacterial and antiviral properties. Specific flavonoids were described to function as antioxidants, enzyme inhibitors, epigenetic modulators or even suppressors in some signaling pathways [72,73].

Over the years, researchers have focused mainly on the antioxidant activity of flavonoids, because, as simple as it seems, it targets an important niche in today’s society: pollution. Everyday people are constantly exposed to radiation, air, water and food pollutants, with all being some of the leading causes of oxidative stress. Basically, cells produce persistently reactive species, such as reactive oxygen species (ROS) and reactive nitrogen species (RNS), that can cause many cardiovascular and neurodegenerative diseases as well as cancer and metabolic diseases [74]. Antioxidants are specific compounds that protect the cells against the damaging effects of free radicals [75,76].

Flavonoids are the best-known phytochemicals that act as free radicals scavengers and metal chelators [77]. This important property of flavonoids has been the subject of several studies in past years. Although there are plenty of in vitro studies that demonstrate that flavonoids are some of the most important antioxidant molecules in animal cells, the antioxidant efficacy of flavonoids in vivo is less documented due to the poor knowledge about flavonoids’ uptake and bioavailability. However, it is well known that the high daily consumption of flavonoids in the form of vegetables, fruits and beverages may be helpful for scavenging ROS, preventing free-radical damage to biological molecules such as lipids, proteins and DNA [78]. A good example is cranberry extract, which is found to inhibit low-density lipoproteins (LDL) oxidation [79], carcinogenesis [80] and oxidative damage of the vascular endothelium [81], with potential in cancer prevention and therapy.

Since the discovery that free radicals are responsible for a number of pathologies, there has been renewed interest in plant products as a source of natural antioxidants to replace the synthetic ones used in medicine, cosmetics and food. That is the reason why, due to the number of beneficial properties of flavonoids, the aim of this review is also to focus on the latest findings on the potential of flavonoids in cancer prevention.

### 5.1. Flavonoids in Cancer Prevention

Oncology is a vast and complex research field, summing up multiple signaling pathways that cooperate for the purpose of tumor survival and proliferation. It is well known that conventional anticancer methods cause multiple kinds of damage, both to the tumor and to the whole body, so it is necessary to find alternative methods of treatment. These methods must have many characteristics, among which they must have the ability to modulate the cancer cell from different points of view at the same time. Flavonoids appear to have huge potential in preventing and treating cancer cells, since they are shown to have antitumor activity by various mechanisms, including the induction of apoptosis and cell cycle arrest and the suppression of cell growth and proliferation [82].

**Figure 3:**
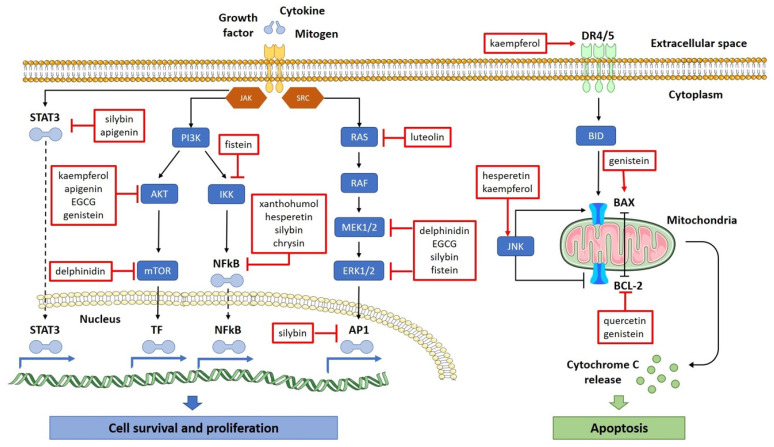
Proposed signaling networks and cell physiological effects mediated by flavonoids.

The list of preventive properties of flavonoids starts with their ability to affect the initiation and promotion stages of carcinogenesis and continues with the capacity to arrest the cell cycle and to induce apoptosis by downregulating proto-oncogenes, upregulating tumor suppressor genes and inhibiting many cancer-triggering factors [83]. Some of the major effects of flavonoids on tumor cells are illustrated in Figure 3.

#### 5.1.1. Pro-Oxidant and Antioxidant Potential

As mentioned earlier, flavonoids are a subclass of polyphenols mainly known for their antioxidant activity within the cells. This is an important feature, because the implications of a high concentration of ROS can jeopardize cellular integrity, leading to oxidative stress, which represents one of the main causes of cancer [84]. The antioxidant activity of flavonoids is possible due to the hydroxyl groups within their structure—structures that are able not only to reduce ROS but also to inhibit ROS-producing enzymes such as superoxide dismutase, cyclooxygenase, xanthine oxidase or NADPH oxidase; and to induce antioxidant enzymes such as UDP-glucuronyltransferase, glutathione-S-transferase or quinone reductase. Furthermore, flavonoids are able to chelate metal atoms such as iron or copper. All of these mechanisms aim to prevent intracellular lipid peroxidation and protein damage but also DNA and RNA instability (which leads to the formation of loss of function mutations in vital genes such as tumor suppressor genes) [85].

Although this is a crucial property, it does not represent the end of the flavonoids’ properties, because all of these antioxidative mechanisms occur only in the normal cells, thus demonstrating their preventive properties. In tumor cells, however, there is a change in the purpose of these phytochemicals: they are not intended to protect the cancer cell, but, on the contrary, the ultimate goal is to destroy them. The mechanisms are still unclear, but there is a probability that some of the flavonoids, such as luteolin and apigenin, can exert pro-oxidative functions due to glutathione depletion and the inhibition of the superoxide dismutase in several cancer cell lines [85].

#### 5.1.2. DNA Protection and Depletion

It was already noticed that flavonoids have the ability to protect genetic material from inducing potential mutations, but again, this property is only applicable in self-cells. In tumoral cells, flavonoids manage to induce DNA depletion, along with modulating the level of gene expression, which fundamentally represent pro-apoptotic and anti-inflammatory mechanisms. As shown in Figure 2, some of the flavonoids, such as genistein or hesperetin, can upregulate tumor suppression genes, such as BAX or JNK, or can downregulate proto-oncogenes, such as BCL-2, and these genetic modulations lead to apoptosis induction or the inhibition of cell survival and proliferation [86,87].

Moreover, the molecular mechanisms are much more complex; some of them are still unknown, and some of them are applicable for specific compounds, and only in specific cancer cell lines. However, Table 4 attempts to present a bigger picture based on the broad spectrum of action of flavonoids on multiple cancer cell lines.

Besides in vitro studies, there is some information about flavonoids’ capabilities in in vivo models, but this subject is still in its incipient state. Although mostly carcinogenic animal models have been used, especially mice and hamsters, favorable results have appeared regularly. Due to the encapsulation of the compounds, which leads to the avoidance of complications related to the low bioavailability of flavonoids, the clinical results support the hypothesis that flavonoids may be compounds with real therapeutic impact in the future. One study shows that 8.98 µmol/L of quercetin may lead to the suppression of hyperplastic nodules with minimum preneolastic lesions in the parenchyma of rats with hepatic carcinoma induced by diethylnitrosamine treatment [149]. Another study demonstrated that poly (lactic-co-glycolite) nanoparticles loaded with apigenin induce the intrinsic mode of apoptotic cell death and suppress epidermal hyperplasia in Swiss albino mice [150]. Thus, it is safe to say that flavonoids may have a strong impact on tumor cell manipulation in vivo, but this topic needs further study.

## 6. Conclusions and Future Prospects

Cancer is one of the most controversial and debated subjects regarding human health. Over the years, marked improvements have been made in the search for novel therapies for cancer prevention and/or treatment. Unfortunately, most of the conventional therapies exert harmful side effects or are unaffordable for most patients. Recently, researchers have been focused on finding novel anticarcinogenic agents by investigating naturally occurring bioactive compounds based on the well-known health benefits of various edible plants.

Flavonoids have been shown to possess a variety of health benefits, and many studies suggest that they may be promising candidates in the prevention and treatment of various chronic diseases, including cancer. Their powerful antioxidant activity seems to be key to their therapeutic properties; however, much more work has to be done in order to fully understand their mechanisms of action. Clinical testing should be implemented, especially using nanocarriers loaded with flavonoids such as liposomes, extracellular vesicles, micro-/nanocapsules or emulsions for administration that would target tumor cells in order to draw the bigger picture of the pharmacokinetic processes exerted by flavonoids in the human body. Thus, the paradigm could be changed in terms of the usefulness of these compounds, which could have enormous potential in cancer treatment, not just in prevention. Potential optimal doses for clinical administration could be established with clinical trials. 

This study highlights the anticarcinogenic effects of flavonoids on various cancer cell lines based on their biological effects. Moreover, the contents of these phytochemicals in several fruits, vegetables and spices are presented based on reported data in order to give an overview of some of the richest sources of flavonoids. There are also some unconventional sources of flavonoids based on genetically modified organisms—sources that are little-studied by the scientific community so far but with huge potential in the mass production of these phytochemicals. Interdisciplinary genetic and biochemical techniques may be useful in facilitating the production and use of phytochemicals for therapeutic purposes, but the subject still requires ongoing research.

Although this review focuses on dietary plants that contain high concentrations of flavonoids, it is not permissible to neglect an increasingly important topic—the waste left over from the production of food that is our daily diet. It has been shown that waste still contains high concentrations of biologically active compounds, including flavonoids, so studies to support the recirculation of waste for medical purposes would be desirable.

Undoubtedly, the subject of flavonoids has demonstrated a series of advantages (e.g., antioxidant capacity, plant abundance, versatility of compounds) and disadvantages (e.g., low bioavailability, lack of information on the ability of metabolism and absorption of flavonoids by the human body) in time for the scientific world. The biologically active compounds from plant sources have immense medical potential, but future studies need to be conducted in order to demonstrate the already existing properties of these compounds.

## Figures and Tables

**Table 1:** Flavonoids found in berries and fruits; FW—fresh weight; DW—dry weight.

Source	Subclass	Major Compounds	Conc. mg/100 g FW	Conc. mg/100 g DW	Conc. mg/100 mL	Refs.
Blackberry (*Rubus* spp.)	Flavan-3-ols	(+)-Catechin			0.166–1.029	[24]
		(−)-Epicatechin			0.012–6.14	
	Flavonols	Quercetin			0.39–1.794	[24]
	Anthocyanins	Cyanidin-3-glucoside	57.2 ± 2.5		0.039–2.551	[24,25]
		Cyanidin-3-rutinoside	25.0 ± 2.8		0.192–11.933	
		Cyanidin-3-xyloside	48.3 ± 5.6			[25]
		Delphinidin-3-glucoside	516.5 ± 9.3			
Chokeberry (*Aronia melanocarpa*)	Flavonols	Kaempferol	0.00–0.69			[26]
		Quercetin	8.90–37.46			
	Anthocyanins	Cyanidin	26.95–947.52			[26]
		Delphinidin	0.65			
		Malvidin	1.22			
		Pelargonidin	0.51–1.44			
		Peonidin	0.08			
		Petunidin	2.79			
Blueberry (*Vaccinium angustifolium*)	Anthocyanins	Cyanidin-3-glucoside	5.1 ± 0.9	7.7 ± 0.7		[15,25]
		Delphinidin-3-glucoside	27.3 ± 3.1	47 ± 2.4		
		Malvidin-3-glucoside	1.9 ± 0.8	94.3 ± 4.5		
		Peonidin-3-glucoside	15.1 ± 2.4			
		Petunidin-3-glucoside	28.1 ± 4.1	37.7 ± 1.9		
	Flavan-3-ols	(+)-Catechin	81.8 ± 9.17	43.1 ± 1.9		[15,27]
		(+)-Epicatechin	9.25 ± 0.15			
		Epicatechin gallate	0.48 ± 0.52			
		Proanthocyanins		35 ± 1.3		[15]
	Flavonols	Kaempferol	5.17 ± 0.04			[15,27]
		Kaempferol-3-glucoside	5.45 ± 0.24			
		Quercetin-3-galactoside	0.19 ± 0.09	78.6 ± 1.1		
		Quercetin-3-glucoside	2.38 ± 0.35			
		Quercetin-3-glucuronide	1.76 ± 0.12			
Cherry (*Prunus* spp.)	Flavan-3-ols	(+)-Catechin			0.036–1.117	[24]
		(−)-Epicatechin			0.051–2.406	
		Proanthocyanidins		10.54 ± 0.19		[28]
	Flavonols	Kaempherol-3-rutinoside		0.03 ± 0.00		[28]
		Quercetin			0.026–0.391	[24]
		Quercetin-3-rutinoside		0.09 ± 0.01		[28]
	Anthocyanins	Cyanidin-3-glucoside			0.078–1.207	[24]
		Cyanidin-3- glucosylrutinoside			0.737–36.128	
		Cyanidin-3-rutinoside			0.321–9.144	
Raspberry (*Rubus idaeus*)	Flavan-3-ols	(+)-Catechin		7.4 ± 0.1	0.544–1.540	[15,24]
		(−)-Epicatechin	2.94 ± 1.25	102.4 ± 4	2.165–4.359	[15,24,27]
	Flavonols	Kaempferol-3-glucoside	0.30 ± 0.45			[27]
		Quercetin	0.5		0.196–0.392	[24,29]
		Quercetin-3-glucoside	0.10 ± 0.32			[27]
		Quercetin-3-glucuronide	0.54 ± 0.75			
	Anthocyanins	Cyanidin 3-glucoside	57.5 ± 3.4	74.4 ± 0.8		[15,25]
		Cyanidin-3-glucosylrutinoside	56.4 ± 3.8		0.489–2.529	[24,25]
		Cyanidin-3-rutinoside	19.6 ± 1.2		0.594–1.072	
		Cyanidin-3-sophoroside	0.4 ± 0.1		5.783–12.469	
		Petunidin 3-glucoside	57.5 ± 3.4			[25]
Blackcurrant (*Ribes nigrum*)	Flavonols	Quercetin	3.7 ± 0.1		0.2–0.385	[24,29]
		Kaempferol	0.1 ± 0.1			[29]
	Anthocyanins	Cyanidin-3-rutinoside			1.616–8.877	[24]
		Delphinidin-3-glucoside			0.22–2.674	
		Delphinidin-3-rutinoside			2.404–17.921	
Strawberry (*Fragaria × ananassa*)	Flavan-3-ols	(+)-Catechin	2.51 ± 0.05	45.8 ± 0.5	0.704–0.813	[15,24,27]
		(−)-Epicatechin	6.80 ± 2.20		0.153–0.201	
		Epicatechin gallate	0.45 ± 0.32			[27]
	Flavonols	Isorhamnetin	0.57 ± 0.01			[30]
		Kaempferol	0.28 ± 0.01 0.5 ± 0.3 6.13 ± 0.52			[27,29,30]
		Kaempferol-3-glucoside	1.04 ± 0.28	8 ± 0.1		[15,27]
		Quercetin	0.6 ± 0.5 19.0 ± 2.20		0.031–0.168	[24,27,29]
		Quercetin-3-galactoside	0.35 ± 0.49			[27]
		Quercetin-3-glucoside	0.20 ± 0.48			
		Quercetin-3-glucuronide	3.35 ± 1.58			
	Flavones	Apigenin	0.24 ± 0.01			[30]
	Anthocyanins	Cyanidin 3-rutinoside	0.7 ± 0.1			[25]
		Pelargonidin 3-glucoside	347.8 ± 10.5			
		Pelargonidin 3-rutinoside	52.4 ± 4.8			
		Peonidin 3-rutinoside	7.6 ± 1.4			
Apple (*Malus domestica*)	Flavan-3-ols	(+)-Catechin			0.152–1.523	[24]
		(−)-Epicatechin			0.414–2.591	
	Flavonols	Isorhamnetin	14.42 ± 0.97			[30]
		Kaempferol	5.07 ± 0.71			
		Quercetin	2.0 ± 0.4 5.16 ± 0.32		0.04–0.092	[24,29,30]
	Flavones	Luteolin	1495 ± 45			[30]
Plum (*Prunus* spp.)	Flavonols	Isorhamnetin	5.23 ± 0.3			[30]
		Kaempferol	3.17 ± 0.12			[30]
		Quercetin	1.5 0.34 ± 0.6			[29,30]
		Quercetin 3-rutinoside		15 ± 2		[28]
	Flavones	Luteolin	3.98 ± 0.04			[30]
	Flavan-3-ols	Proanthocyanidins		969 ± 187		[28]
Peach (*Prunus persica*)	Flavonols	Kaempferol	1.43 ± 0.17			[30]
		Kaempherol-3-hexoside		4 ± 1		[28]
		Kaempherol-3-rutinoside		5 ± 1		
		Quercetin 3-rutinoside		6 ± 1		
	Flavones	Luteolin	3.39 ± 0.42			[30]
	Flavan-3-ols	Proanthocyanidins		1379 ± 62		[28]
Grapes (*Vitis vinifera*)	Flavonols	Kaempferol	8.91 ± 0.4 5.35 ± 0.59			[27,30]
		Kaempferol-3-glucoside	0.68 ± 1.2			[27]
		Quercetin	1.19 ± 0.03 0.2			[29,30]
		Quercetin-3-glucoside	0.36 ± 0.48			[27]
		Quercetin-3-glucuronide	3.11 ± 1.54			
	Flavan-3-ols	Catechin	1.44 ± 0.09			[27]
		Epicatechin	2.02 ± 1.17			
		Epicatechin gallate	0.29 ± 0.30			
Orange (*Citrus × sinensis*)	Flavonols	Isorhamnetin	0.87 ± 0.08			[30]
		Kaempferol	0.51 ± 0.05			
		Quercetin	0.17 ± 0.02			
	Flavones	Luteolin	0.45 ± 0.04			[30]
		6,8-di-C-Glu-Apigenin			4.15–8	[31]
	Flavanones	Hesperetin	31 ± 2		3.51–55.2	[29,31]
		Naringenin	11 ± 2			[29]
Cranberry (*Vaccinium* spp.)	Flavonols	Myricetin	23			[29]
		Quercetin	16			
Grapefruit (*Citrus* × *paradisi*)	Flavonols	Kaempferol	0.4 ± 0.1			[29]
		Quercetin	0.5 ± 0.1		0.19	[29,31]
	Flavanones	Hesperetin	1.5 ± 0.3		0.25–1.79	[29,31]
		Naringenin	53 ± 6		0.98–8	
		Narirutin			2.5–17	[31]
Lemon (*Citrus limon*)	Flavanones	Hesperetin	17		3.84–41	[29,31]
		Naringenin	0.5			[29]
	Flavanones	6,8-di-C-Glu-Apigenin			1–1.45	[31]
		6,8-di-C-Glu-Diosmetin			4.05–5.8	
		7-*O*-Rut-Luteolin			1.5–6.5	
Apricot (*Prunus* spp.)	Flavonols	Kaempferol	0.38 ± 0.05 5.44 ± 0.12			[32,33]
		Kaempherol-3-rutinoside	0.03			[28]
		Myricetin	0.69 ± 0.07			[32]
		Quercetin	4.31 ± 0.07			[33]
		Quercetin-3-*O*-glucoside	7.57 ± 2.87			[32]
		Quercetin 3-rutinoside		0.23 ± 0.01		[28]
		Rutin	3.77 ± 0.05	0.16–0.26		[33,34]
	Flavan-3-ols	Catechin		3.14		[35]
		Proanthocyanidins		3.04 ± 0.08		[28]
	Flavones	Apigenin	0.22 ± 0.01			[33]
		Apigenin 7-*O*-glucoside	60.47 ± 1.08			
		Luteolin	0.68 ± 0.42			[32]
		Luteolin 7-xyloside	4.60 ± 0.02			[33]
	Anthocyanins	Cyanidin 3-(4″-acetylrutinoside)	56.71 ± 1.13			[33]
		Cyanidin 3-(6″-acetylglucoside)	11.34 ± 0.16			
		Cyanidin 3-*O*-galactoside	4.13 ± 0.05			
		Cyanidin 3-rutinoside	4.47 ± 0.09			
		Petunidin 3-galactoside	6.61 ± 0.05			
		Petunidin 3-rutinoside	2.80 ± 0.05			

**Table 2:** Flavonoids found in vegetables; FW—fresh weight; DW—dry weight.

Source	Subclass	Major Compounds	Conc. mg/100 g FW	Conc. mg/100 g DW	Refs.
Onion (*Allium cepa*)	Flavonols	Isorhamnetin-4′-glucoside	5.398 ± 0.042		[48]
		Kaempferol	4.13 ± 0.24		[30]
		Quercetin	1.42 ± 0.06		
		Quercetin-3,4′-diglucoside	29.646 ± 0.005	171.34 ± 0.13	[48,49]
	Flavones	Apigenin	2.62 ± 0.12		[30]
	Anthocyanins	Cyanidin-3-(6″-malonylglucoside)	1.718 ± 0.075		[48]
		Peonidin-3′-glucoside		0.19	[49]
Kale (*Brassica oleracea* var.)	Flavonols	Isorhamnetin	5.98 ± 0.41		[30]
		Kaempferol	2.4 ± 0.23		
		Quercetin	0.48 ± 0.03		
	Flavones	Apigenin	0.28 ± 0.02		[30]
		Luteolin	2.39 ± 0.2		
Celery (*Apium graveolens*)	Flavones	Apigenin	13.93 ± 0.52 0.461	79.42 ± 0.77	[30,50,51]
		Apigenin-7-*O*-glucoside		156 ± 7	[52]
		Luteolin	2.31 ± 0.11 0.088	62.43 ± 0.59	[30,50,51]
		Luteolin-7-*O*-glucoside		654 ± 8	[52]
	Flavonols	Kaempferol	0.46 ± 0.03	1.06 ± 0.03	[30,51]
		Myricetin	105.05 ± 4.46		[53]
		Rutin	13.99 ± 0.58		
		Quercetin	5.31 ± 0.21		
	Flavan-3-ols	Epicatechin	8.90 ± 0.42		[53]
Chili pepper (*Capsicum* var.)	Flavonols	Isoquercetin	1.742 ± 0.055		[54]
		Kaempferol-3-glucoside	3.479 ± 0.02		
		Myricetin	2.388 ± 0.06		
		Quercetin	0.16 ± 0.02		[30]
	Flavones	Apigenin	0.5		[30]
		Luteolin	2.54 ± 0.05		
Radish (*Raphanus raphanistrum* subsp. *sativus*)	Flavonols	Kaempferol	3.23 ± 0.44		[30]
		Quercetin	0.52 ± 0.07		
	Flavones	Apigenin	0.22 ± 0.03		[30]
		Luteolin	1.95 ± 0.27		
Soybean (*Glycine max*)	Flavonols	Quercetin	0.17		[30]
	Flavones	Luteolin	0.94 ± 0.12		[30]
Spinach (*Spinacia oleracea*)	Flavonols	Kaempferol	0.89 ± 0.04		[30]
Cabbage (*Brassica oleracea*)	Flavonols	Kaempferol	3.12 ± 0.02	11.0 ± 0.8	[30,55]
		Quercetin	0.49	16.1 ± 1.0	
	Flavones	Luteolin	3.27 ± 0.02		[30]
	Anthocyanins	Cyanidin-3,5-*O*-diglucoside	3.2		[56]
		Cyanidin-3-(feruloyl)-diglucoside-5-glucoside	7.3		
		Cyanidin-3-(sinapoyl)-*O*-diglucoside-5-*O*-glucoside	2.7		
		Cyanidin-3-coumaroyl-dihexoside-5-hexoside	9.4		
Broccoli (*Brassica oleracea* var. *italica*)	Flavonols	Kaempferol	211 ± 6		[30]
		Quercetin	0.53 ± 0.03		

**Table 3:** Flavonoids found in spices; FW—fresh weight; DW—dry weight.

Source	Subclass	Major Compounds	Conc. mg/100 g FW	Conc. mg/100 g DW	Ref.
Celery (*Apium graveolens*)	Flavones	Apigenin-7-*O*-glucoside		156 ± 7	[52]
		Luteolin-7-*O*-glucoside		654 ± 8	
Cumin (*Cuminum cyminum*)	Flavones	Apigenin-7-*O*-glucoside		146 ± 2	[52]
		Luteolin-7-*O*-glucoside		224 ± 7	
Dill (*Anethum graveolens*)	Flavonols	Isorhamnetin	15–72		[58]
		Kaempferol	16–24		
		Quercetin	48–110		
Oregano (*Origanum vulgare*)	Flavones	Apigenin	2–4		[58]
		Apigenin-7-*O*-glucoside		254 ± 1	[52]
		Luteolin	0–3		[58]
		Luteolin-7-*O*-glucoside		301 ± 1	[52]
Fennel (*Foeniculum vulgare*)	Flavones	Apigenin-7-*O*-glucoside		43 ± 1	[52]
		Luteolin-7-*O*-glucoside		211 ± 4	
Cress (*Lepidium sativum*)	Flavonols	Isorhamnetin	1		[58]
		Kaempferol	13		
Basil (*Ocimum basilicum*)	Flavones	Apigenin-7-*O*-glucoside		18	[52]
		Luteolin-7-*O*-glucoside		127 ± 1	
Marjoram (*Origanum majorana*)	Flavones	Apigenin-7-*O*-glucoside		83 ± 3	[52]
		Luteolin-7-*O*-glucoside		461 ± 7	
Chives (*Allium schoenoprasum*)	Flavonols	Isorhamnetin	5		[58]
		Kaempferol	12		
		Quercetin	3		
Parsley (*Petroselinum* *crispum*)	Flavones	Apigenin	0.44 ± 0.01		[30]
		Apigenin-7-*O*-glucoside		752 ± 17	[52]
		Luteolin	1.42 ± 0.03		[30]
		Luteolin-7-*O*-glucoside		125 ± 8	[52]
	Flavonols	Isorhamnetin	1.12 ± 0.1		[30]
		Kaempferol	1.85 ± 0.03		
		Myricetin	151.03 ± 6.68		[53]
		Quercetin	0–1 0.5 ± 0.01 71.33 ± 2.19		[30,53,58]
		Rutin	4.32 ± 0.23		[53]
	Flavan-3-ols	Epicatechin	2.67 ± 0.11		[53]
Thyme (*Thymus vulgaris*)	Flavones	Apigenin	5		[58]
		Apigenin-7-*O*-glucoside		16	[52]
		Luteolin	51		[58]
		Luteolin-7-*O*-glucoside		104 ± 2	[52]
Lovage (*Levisticum officinale*)	Flavonols	Kaempferol	7		[58]
		Quercetin	170		
Coriander (*Coriandrum sativum*)	Flavonols	Quercetin	5		[58]
Rosemary (*Rosmarinus officinalis*)	Flavones	Apigenin-7-*O*-glucoside		50 ± 1	[52]
		Luteolin	4		[58]
		Luteolin-7-*O*-glucoside		71 ± 2	[52]
Mint (*Mentha* var.)	Flavones	Apigenin	18–99		[58]
		Luteolin	11–41		
Sage (*Salvia officinalis*)	Flavones	Apigenin-7-*O*-glucoside		53 ± 1	[52]
		Luteolin-7-*O*-glucoside		495 ± 1	
Watercress (*Nasturtium officinale*)	Flavonols	Kaempferol	1		[58]
		Quercetin	4		[58]
Cinnamon (*Cinnamomum* var.)	Flavan-3-ols	Proanthocyanins		8960	[28]
Tarragon (*Artemisia dranunculus*)	Flavonols	Isorhamnetin	5		[58]
		Kaempferol	11		
		Quercetin	10		
	Flavones	Luteolin	1		

**Table 4:** Biological activities of flavonoids; ↑—induction, activation, upregulation, elevation; ↓—suppression, inactivation, downregulation, block.

Cancer Type	Cell Line	Compound	Conc.	Main Biological Effects	Ref.
Skin cancer	A431, SCC-13	Fisetin	0–80 µM	↑ Apoptosis ↑ Cell cycle arrest at G2/M phase ↓ Cell viability ↓ Colony formation ↓ Δψm	[88]
	A375	Luteolin	0–80 µM	↑ Apoptosis ↑ Cell cycle arrest at G0/G1 phase ↓ Colony formation ↓ Cell proliferation	[89]
	B16F10	Galangin	0–100 µmol/L	↑ Phosphor-p-38 MAPK ↑ Apoptosis ↓ Δψm ↓ Cell viability	[90]
	SK-MEL-5, SK-MEL-28	Silybin	0–80 µM	↑ Cell cycle arrest at G1 phase ↓ Cell viability ↓ Cell proliferation ↓ Kinase activity of MEK1/2 and RSK2 ↓ Expression of NF-κB, Ap-1 and STAT3 ↓ Phosphorylation of ERK1/2 and RSK2	[91]
	A375, RPMI-7951, Hs294T	Fisetin	0–20 µM	↓ Cell invasion ↓ Phosphorylation of MEK1/2 and ERK1/2 ↓ Activation of IKK ↓ Activation of the NF-κB signaling pathway	[92]
	B16-F10	Anthocyanins	0–500 µg/mL	↓ Cell proliferation	[93]
			0–800 μg/mL	↑ Cell cycle arrest at G0/G1 phase ↑ Apoptosis ↓ Cell viability ↓ Cell proliferation	[94]
	B16-F1	Anthocyanins	0–1 mg/mL	↓ Cell growth ↓ Cell migration ↓ Tube formation ↓ Expression of MMP-2/-9 and VEGF ↓ Angiogenesis	[95]
	A431	Resveratrol + ALA-PDT therapy	0–120 mg/mL	↑ Apoptosis ↑ MAPK pathway ↓ Cell proliferation	[96]
	A375.S2	Chrysin	0–15 µM	↑ Cell morphological changes ↓ Cell viability ↓ Cell migration and invasion ↓ Expression of MMP-2 ↓ Expression of NF-κB p65	[97]
Breast cancer	MDA-MB-231, MCF-7	Luteolin	0–100 µM	↓ Cell viability ↓ Cell migration ↓ Expression of Notch-1, Hes-1, Hey, VEGF, Cyclin D1 and MMP -Regulating miRNAs	[98]
	MCF-7, MDA-MB-231	Epigallocatechin-3-gallate	0–40 µM	↑ TIMP -3 levels↓ Cell proliferation by restoring the MP/TIMP balance	[99]
	MCF-7	Hesperetin	0–200 µM	↑ ROS generation ↑ ASK1/JNK pathway ↑ Apoptosis ↓ Δψm	[87]
	BT-474	Apigenin	0–100 µM	↑ Apoptosis ↓ STAT3 signaling ↓ Cell proliferation ↓ Chlorogenic survival	[100]
	MCF-7	Kaempferol	0–100 µM	↑ Extracellular lactate levels ↓ Cell proliferation ↓ Glucose uptake	[101]
			0–100 mg/mL	↑ Apoptosis ↓ Cell proliferation ↓ Δψm	[102]
	MDA-MB-231	Isorhamnetin	0–40 µM	↓ Cell proliferation ↓ Cell migration ↓ Cell adhesion ↓ Expression of MMP-2 and MMP-9	[103]
	MDA-MB-231, MDA-MB-468	Quercetin	0–100 µM	↓ Cell proliferation ↓ Cell viability ↓ β-Catenin	[104]
	MDA-MB-231 (4175) LM2, MDA-MB-435	Luteolin	0–100 µM	↑ Apoptosis ↓ Cell migration ↓ Cell viability ↓ VEGF secretion	[105]
	MDA-MB-231	Luteolin	0–40 µM	↑ Apoptosis ↓ Cell viability ↓ Expression of MMP-9 ↓ Cell migration ↓ Cell invasion	[106]
	MDA-MB-453, MCF-7	Luteolin	10 µM	↑ Apoptosis ↑ Expression of miR-203 ↓ Cell viability ↓ Ras/Raf/MEK/ERK signaling pathways	[107]
	MDA-MB-231, MCF-7, MDA-MB-453	Delphinidin	40 μmol/L	↓ Cell viability ↓ Cell proliferation ↓ Cell migration ↓ Wnt/β-catenin signaling pathway -Modulating miR-34a and HOTAIR	[108]
	MCF-7	Quercetin	25 μmol/mL	↑ Apoptosis ↑ ROS levels and MDA ↓ Cell viability ↓ Cell proliferation ↓ Antioxidant enzymes activity	[109]
Ovarian cancer	ES2	Delphinidin	0–100 µM	↑ Apoptosis ↓ Cell proliferation ↓ Cell migration ↓ AKT, ERK1/2, and MAPK signaling pathways	[110]
	SK-OV-3	Genistein	0–90 µM	↑ Apoptosis ↓ Cell proliferation ↓ Δψm	[111]
	OVCAR-3, SKOV-3	Kaempferol	0–100 µM	↑ Apoptosis ↑ Expression of DR4, DR5, CHOP, JNK, ERK1/2, p38 ↓ Cell proliferation -Modulates the expression of apoptotic pathway proteins	[112]
	CAOV3	Quercetin	0–100 µM	↑ Apoptosis ↓ Cell viability	[113]
	A2780/CP70, OVCAR-3	Kaempferol	0–50 µM	↑ Cell cycle arrest at G2/M phase via Chk2 ↑ Apoptosis via death receptors ↓ Cell viability	[114]
	PA-1	Quercetin	0–200 µM	↑ Apoptosis ↓ Cell viability ↓ Bcl-2, Bcl-xL	[115]
	A2780, OVCAR-3, SKOV-3	Apigenin Luteolin Myricetin	0–100 µM	↑ ROS levels ↑ MDA levels ↑ Apoptosis ↑ Cell cycle arrest at G0/G1 and G2/M phase ↓ Cell viability	[116]
Cervical cancer	HeLa	Quercetin	0–100 µM	↑ Apoptosis ↑ Cell cycle arrest at G2/M phase ↑ ROS levels ↓ Cell proliferation ↓ Δψm	[117]
	HeLa	Kaempferol	0–100 mg/mL	↓ Cell proliferation	[102]
			2.5–100 µM	↑ Bax ↓ Expression of Cyclin B1 ↓ Expression of CDK1 ↓ NF-κB nuclear translocation ↓ Bcl-2	[6]
			0–100 µM	↑ Apoptosis ↓ Cell viability ↓ PI3K/AKT and hTERT pathways	[118]
	SiHa	Kaempferol	0–100 µg/mL	↑ Apoptosis ↑ Intracellular free Ca^2+^ ↓ Cell proliferation ↓ Δψm	[119]
Lung cancer	H446	Genistein	0–100 µM	↑ Apoptosis ↑ Cell cycle arrest at G2/M phase ↓ Cell proliferation ↓ Cell migration	[120]
	NCI-H1299, -H460	Luteolin	0–50 µM	↑ Apoptosis ↓ Cell viability	[121]
	A549	Kaempferol	0–50 µM	↓ Cell proliferation ↓ Cell migration ↓ TGF-β1-induced EMT	[122]
			0–100 mg/mL	↓ Cell proliferation	[102]
	RAW 264.7	Luteolin	0–30 µM	↓ Cell proliferation ↓ Cell migration ↓ STAT6 phosphorylation and the TAM phenotype ↓ Expression of CCL2 and migration of monocytes	[123]
	A549	Genistein	0–200 µM	↑ Apoptosis ↑ Bax mRNA level ↑ Expression of miR-27a ↓ Cell proliferation ↓ Cell viability ↓ Bcl-2 mRNA level ↓ Expression of MET protein	[124,125]
	A549	Apigenin	0–100 µM	↓ Cell proliferation ↓ Cell migration and invasion by targeting the PI3K/Akt signaling pathway	[126]
	A549, H1299	Daidzein	0–80 µmol/L	↑ Apoptosis ↓ Cell proliferation	[41]
	A549	Delphinidin	0–80 µM	↓ Cell proliferation ↓ ERK, mTOR and p70S6K signaling pathways	[127]
	A549	Kaempferol	0–50 µM	↑ Apoptosis ↑ Expression of miR-340 ↓ Cell proliferation ↓ Cell viability ↓ Expression of Cyclin D1 ↓ p-PI3K and p-AKT levels	[128]
	A549	Fisetin	0–40 µM	↑ Apoptosis ↑ Cell cycle arrest at G2/M phase ↓ Cell viability ↓ Cell proliferation ↓ Cell adhesion ↓ Cell invasion ↓ Cell migration ↓ ERK signaling pathway via MEK1/2	[129]
	H1299, A549	Epigallocatechin-3-gallate	0–40 µM	↑ Apoptosis ↓ Cell proliferation ↓ Expression of p-PI3K and p-Akt	[130]
	A549	Hesperetin	0–100 µM	↓ Cell proliferation	[9]
	A549	Epigallocatechin-3 -gallate	40 µM	↑ miR-155 ↑ Cell cycle arrest at G0/G1 phase ↓ Cell proliferation ↓ miR-212	[131]
Colon cancer	HT-29	Kaempferol	0–60 µmol/L	↑ Apoptosis ↓ Δψm	[132]
	HT-29	Epigallocatechin-3-gallate	0–50 µM	↑ MAPK and Akt signaling pathways ↓ p38 and ERK1/2 signaling pathways	[133]
	HCT-116	Resveratrol	0–150 µM	↑ Apoptosis ↑ DNA damage	[134]
	HCT-116, SW480, LoVo, HT-29	Naringenin	0–200 µM	↑ Apoptosis ↓ Cell viability	[135]
	HCT-116, LoVo	Genistein	0–100 µM	↑ Apoptosis ↑ Bax mRNA level ↓ Cell proliferation ↓ Cell viability ↓ Phosphorylation of Akt	[136]
Liver cancer	HepG2, Huh-7, HA22T	Naringenin	0–100 µM	↓ Cell proliferation ↓ TPA-induced cancer cell proliferation	[137]
	Huh-7, HepG2, Hep3B, SK-Hep-1	Xanthohumol	0–15 µM	↑ Apoptosis ↓ Cell viability ↓ Colony forming ↓ Notch1 signaling	[138]
	HepG2	Xanthohumol	0–40 µM	↓ cell proliferation ↑ Apoptosis -modulates NK-kB/p53 signaling pathways	[139]
	Hepa1-6	Genistein	0–100 µM	↑ Apoptosis ↓ Cell viability ↓ Cell proliferation	[140]
	HepG2	Kaempferol	0–100 µM	↑ Apoptosis ↓ Cell proliferation ↓ Cell migration ↓ Cell invasion ↓ Expression of miR-2I	[141]
Prostate cancer	PC-3	Hesperetin	0–120 µM	↑ Apoptosis ↓ Cell proliferation ↓ NK-kB signaling pathway	[142]
	LNCaP	Kaempferol-3-*O*-rhamnoside	0–926 µM	↑ Apoptosis ↓ Cell proliferation	[143]
	PC-3, DU145	Resveratrol	0–100 µM	↑ Autophagy cell death	[144]
Gastric cancer	SGC-7901, MKN28	Kaempferol	0–200 µM	↑ Apoptosis ↑ Cell cycle arrest at G2/M phase ↓ Cell proliferation ↓ Cell viability	[145]
	HGC-27, SGC-7901	Apigenin	0–20 µg/mL	↑ Apoptosis ↓ Cell proliferation ↓ Δψm	[146]
	SGC-7901, MGC-803, HGC-27	Hesperetin	0–400 µM	↑ Apoptosis ↓ Cell proliferation ↓ Δψm ↓ Cell viability ↓ ROS levels	[147]
	HGC-27, SGC-7901	Myricetin	0–40 µM	↑ Apoptosis ↑ Cell cycle arrest at G2/M phase ↓ Cell proliferation	[148]
	SCG-7901	Kaempferol	0–100 mg/mL	↓ Cell proliferation	[102]

## Data Availability

Not applicable.

## Abbreviations

DW	Dry weight
RE	Rutin equivalent
FW	Fresh weight
CE	Catechin equivalents
PAP1/AtMYB75	Production of anthocyanin product 1/*A. Thaliana*’s MYB transcription factor 75
cDNA	Complementary DNA
ANS	Anthocyanidin synthase
MAS	Mannopine synthase
Pd35S	Cauliflower mosaic virus double 35S promoter
CHI	Chalcone isomerase
FNS	Flavone synthase
Tnos	*Agrobacterium tumefaciens nos* terminator
F3H	Flavanone-3-hydroxylase
CRISPR	Clustered regularly interspaced short palindromic repeats
Cas	CRISPR-associated proteins
IFS	Isoflavone synthase
SMV	Soya bean Mosaic virus
ROS	Reactive oxygen species
RNS	Reactive nitrogen species
LDL	Low-density lipoprotein
